# Perfusion image guided mechanical thrombectomy combined with tirofiban successfully revascularize systemic lupus erythematosus related acute large vessel occlusion: A case report

**DOI:** 10.1097/MD.0000000000025779

**Published:** 2021-05-07

**Authors:** Han Wang, Haitao Hu, Jing Xu, Cong Qian

**Affiliations:** a Department of Geriatrics, Tongde Hospital of Zhejiang Province; b Department of Neurology; c Department of Neurological Surgery, the Second Affiliated Hospital, Zhejiang University School of Medicine, Hangzhou, Zhejiang Province, China.

**Keywords:** acute ischemic stroke, large vessel occlusion, mechanical thrombectomy, perfusion image, systemic lupus erythematosus, tirofiban

## Abstract

**Rationale:**

Systemic lupus erythematosus (SLE) is an important cause of stroke, more than a half the cases present as acute ischemic stroke. Thrombolysis is an effective choice in most cases, but for large vessel occlusion, mechanical thrombectomy is more effective. Here we reported a case of SLE-related stroke with left middle cerebral artery (MCA) occlusion, who was successfully treated by MT and tirofiban.

**Patient concern:**

A 38-year-old female suffered from right hemiplegia and aphasia for 8 hours. She was diagnosed with SLE 20 years ago, and neuropsychiatric SLE was considered 8 months before this onset. One month ago, glucocorticoids were discontinued by herself because of deterioration of bilateral femoral head osteonecrosis.

**Diagnosis:**

Left MCA occlusion was confirmed by computed tomography perfusion.

**Intervention:**

Immediate mechanical thrombectomy was performed and tirofiban was given to prevent re-occlusion of left MCA. Twenty fourhours later oral antiplatelet was given after intracranial hemorrhage was ruled out.

**Outcomes:**

Her neurological symptom improved several days later, and she was transferred to further rehabilitation. At 4 months follow-up she can live independently with mild hypophrasia. There was no further events of ischemic stroke in 1-year follow-up.

**Lessons:**

Mechanical thrombectomy is a highly effective and indispensable treatment for SLE related large vessel occlusion. In addition, tirofiban may reduce vessel reocclusion in special cases such as SLE and artery stenosis.

## 1 Introduction

Systemic lupus erythematosus (SLE) is an important cause of stroke, the prevalence is 3.1% in SLE patients and incidence is 1.25 per 1000 annually, more than half the cases are acute ischemic stroke (AIS). Thrombolysis is an effective and safe choice, but it is not recommended when patients arrive beyond the window period. Nowadays if these patients met the DAWN and DEFUSE 3 eligibility criteria, mechanical thrombectomy (MT) may be an effective treatment. Tirofiban is a nonpeptide selective glycoproteinIIb/IIIa receptor inhibitor and widely used to prevent reocclusion after MT.

Here we reported a case of SLE-related stroke with left middle cerebral artery (MCA) occlusion, who was successfully treated by MT and tirofiban. The informed consent was signed by patient for publication.

## 2 Case presentation

A 38-year-old woman suffered sudden right hemiplegia with aphasia 8 hours ago and was transported to our hospital in June 2018. She was diagnosed with SLE 20 years ago, and neuropsychiatric SLE was considered 8 months before this onset. One month ago, glucocorticoids were discontinued by herself because of deterioration of bilateral femoral head osteonecrosis. Three days before this onset, patient reported transient aphasia for 15 minutes and another aphasia lasted for about 20 minutes 1 day before. She did not go to any medical institution for further assessment until this attack. She had no history of cancer, addictive drug use or oral contraceptive use. On admission, this patient was alert and oriented, vital signs was normal. On physical examination right central facial palsy and right hemiplegia was present with a National Institute of Health Stroke Scale (NIHSS) score of 13. Both complete blood cell count and coagulation parameters were normal. Serum fibrinogen (4.84 g/L) and D-dimer levels (1200 μg/L) were increased. No other abnormal laboratory result was present.

A non-contrast computed tomography (CT) was normal with Alberta stroke program early CT score of 10, but computed tomography angiography (CTA) revealed occlusion of the left middle cerebral artery (MCA). The computed tomography perfusion showed that the volume of penumbra and core infarct volume was 53 mL and 13 mL respectively, and the mismatch ratio was 4.1. AIS with large vessel occlusion (LVO) was diagnosed, intravenous alteplase was not recommended since onset was more than 4.5 hours.

**Figure 1:**
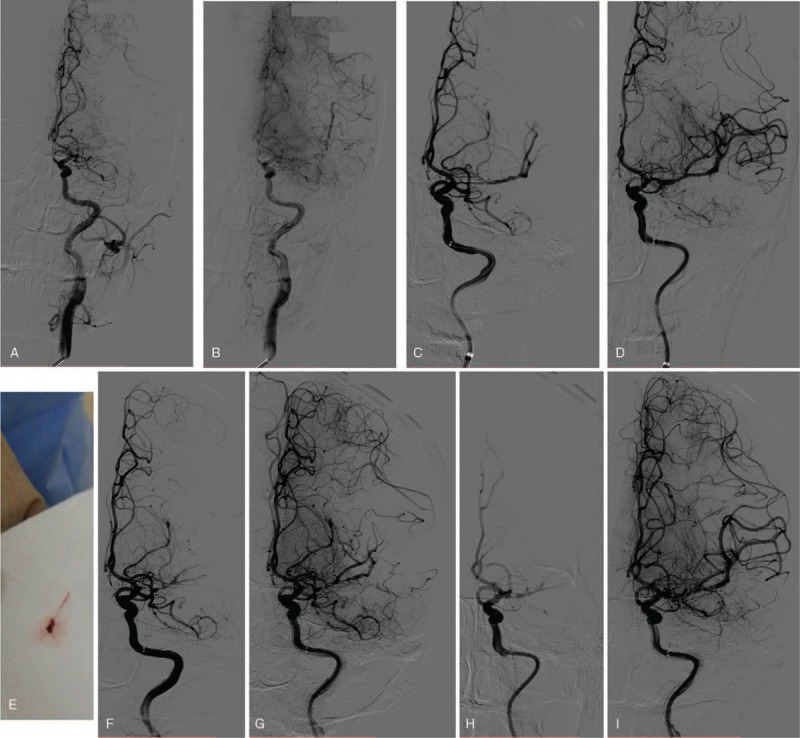
The DSA image before and after mechanical thrombectomy. A and B, left internal carotid angiography showed left M1 occlusion and left anterior cerebral artery supplied the collateral blood flow to middle cerebral artery territory. C, after solitaireFR 6 × 30 mm was deployed, angiography showed normal cerebral blood flow was restored. D, a successful first-pass effect of modified thrombolysis in cerebral infraction 3 was obtained. E, a small amount of clot was removed out of the vessels. F and G, 10 minutes later a second angiography showed the blood flow of left MCA worsened obviously and reocclusion was considered. H and I, 10 minutes after intravenous tirofiban another cerebral angiography showed normal blood flow of left MCA.

Therefore, this patient met both DAWN and DEFUSE 3 eligibility criteria, and according to the updated guidelines, mechanical thrombectomy (MT) was recommended. This patient was sent to neurointerventional suite for revascularization within 60 minutes after arriving at our hospital. The angiography of left common carotid artery showed occlusion of M1 segment of left MCA and left anterior cerebral artery supplied the collateral blood flow to MCA territory at late arterial stage (Fig. 1 A, B). A SolitaireFR 6 × 30 mm (Covidien) was deployed through Rebar18 microcatheter and angiography showed normal cerebral blood flow was restored. After first thrombectomy a small amount of clot was removed from the vessels (Fig. 1 E), a grade 3 of modified thrombolysis in cerebral infraction was restored (Fig. 1 C, D). Ten minutes later a second angiography showed the blood flow of left MCA worsened obviously (Fig. 1 F, G). Tirofiban was continuously given at the speed of (400 μg/h) after an intravenous bolus of 500 μg. Ten minutes later another cerebral angiography showed normal blood flow of left MCA (Fig. 1 H, I). Patient was transferred to neurological ward for further treatment.

**Figure 2:**
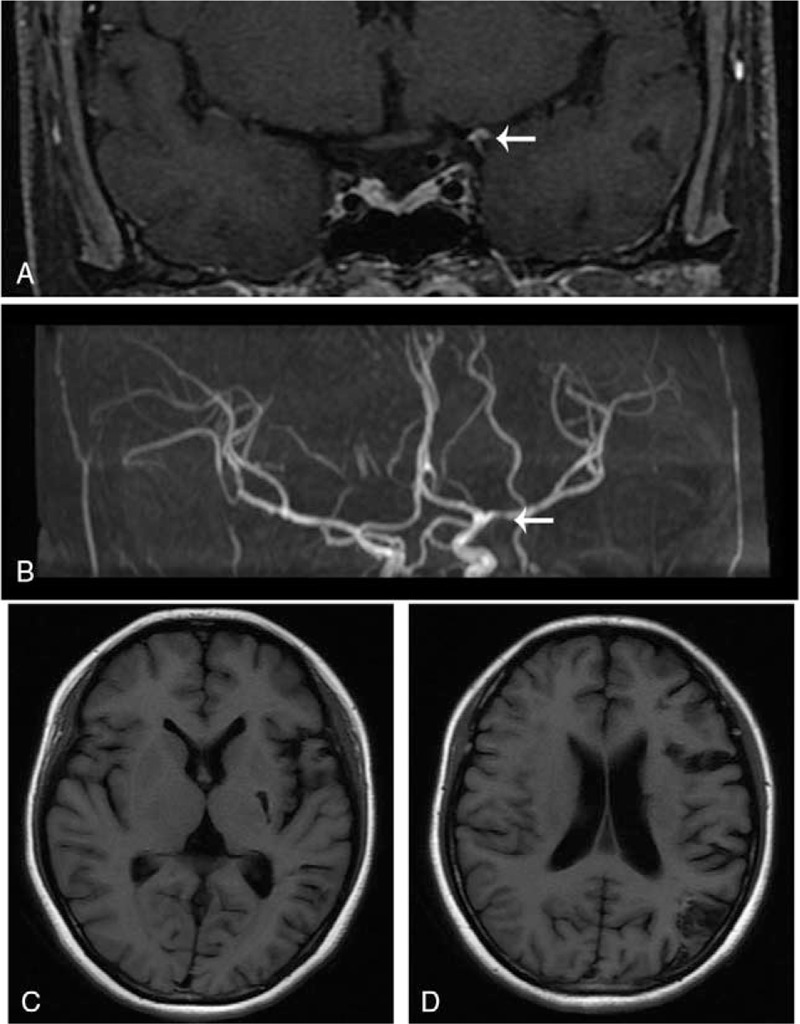
The MR image after MT. A, On the thin slice contrast-enhanced MR an obvious enhancement of vessel wall at left M1 segment was detected. B, MRA revealed stenosis at the same site. C and D, her T1 weighted MR showed multiple encephalomalacia foci 4 months later.

Twenty four hours later oral antiplatelet was given after intracranial hemorrhage was ruled out. Her erythrocyte sedimentation rate increased mildly (26 mm/h). Further lab examination demonstrated her antinuclear antibody (ANA) (1:80) and anti-double-stranded DNA antibody was positive (140.4 IU/mL), these examinations were normal (ANA negative and anti-ds DNA 75.4 IU/mL) 8 months ago. Although her antiphospholipid antibody was negative, a SLE-related ischemic stroke was diagnosed. 40 mg methylprednisolone was daily given orally and intravenous immunoglobulin was refused by the patient. On the thin slice contrast-enhanced magnetic resonance (MR) an obvious enhancement of vessel wall at left M1 segment was detected comparing with the contralateral vessels (Fig. 2 A), and magnetic resonance angiography (MRA) revealed stenosis at the same site (Fig. 2 B). Her neurological symptom improved with NIHSS score of 7 several days later. She was transferred for further rehabilitation.

At 4 months follow-up she can live independently with mild hypophrasia (modified rankin scale scored 1). Her T1 weighted MR showed multiple encephalomalacia foci (Fig. 2 C, D). She continuously took 75 mg clopidogrel and 5 mg methylprednisolone daily and no ischemic stroke was observed in the 1 year follow-up.

## 3 Discussion

AIS is a leading cause of death and morbidity in the world,^[1]^ SLE increases the risk of ischemic stroke especially in the people under age of 50.^[2,3]^ Stroke attacks within the first year or more than 10 years after SLE diagnosis as a bimodal presenting.^[4]^ Secondary antiphospholipid syndrome (APS) was identified in 54% of SLE-related AIS^[4]^ and lifelong anticoagulation is recommended,^[5]^ but anticoagulation was not suitable for this patient due to negative APL. The stenosis of MCA was confirmed (Fig. 2 B) and antiplatelet after 24 hours was recommended according to the guideline.

The medical history, NIHSS score, perfusion image, and cerebral angiography marked the patient's eligibility for DEFUSE 3 trial. This was the first case of SLE-related ischemic stroke treated by MT and she lives independently (modified rankin scale scored 1). The outcome of AIS improved a lot since the intervention of alteplase, which is also widely used in SLE-related AIS and prevent re-occlusion.^[6–8]^ With the improvement of stent retriever, endovascular treatment was proved to be effective since MR CLEAN trial, and MT is the first-line treatment for the AIS patients with large vessel occlusion (LVO).^[9]^ With the advances in imaging, the paradigm shift of early management of AIS was changing from “time is brain” to “image is brain.”^[10]^ Now the time window of carefully selected patients by perfusion image extend to 9 hour,^[11]^ but the short time window of intravenous thrombolysis often causes the patients to miss the opportunity of intravenous thrombolysis and become permanent disabled. In patients in whom the onset extend to 6 hours, but when they meet the eligibility of the criteria of DAWN trial or DEFUSE 3 trial, MT is also recommended by the updated guideline.^[12]^

Successful MT improves the outcome of AIS patients, but early reocclusion after successful MT is an independent predictor of poor outcome and is associated with residual embolic fragments or stenosis.^[13,14]^ In present case early reocclusion occurred 10 minutes after first successful MT. Intravenous tirofiban, a fast-acting non-peptide glycoprotein IIb/IIIa with high selectivity and short half-life, improved the blood flow avoiding another MT. Recently the mounting evidences suggested tirofiban combined with MT is safe and effective^[15–18]^ even before pre-MT is performed.^[19]^ Tirofiban can prevent reocclusion and improve the outcome especially for intracranial artery stenosis related AIS.^[20,21]^ Now the recommendation of tirofiban combined with MT is uncertain, further randomized controlled trials (RCTS) are necessary to clarify this issue.

## 4 Conclusion

SLE related stroke should be taken seriously; rapid diagnosis and treatment including thrombolysis or MT can give patients good outcome. Perfusion image guided MT is an effective and safe method for LVO patient who is out of window period of thrombolysis. Tirofiban can rescue reocclusion after MT especially in patient with artery stenosis and SLE. Lifelong antiplatelet is reasonable for SLE patient with cerebral artery stenosis or AIS.

## Author contributions

**Conceptualization:** Han Wang, Cong Qian.

**Data curation:** Han Wang, Haitao Hu, Jing Xu, Cong Qian.

**Funding acquisition:** Cong Qian.

**Investigation:** Haitao Hu.

**Methodology:** Han Wang, Jing Xu, Cong Qian.

**Supervision:** Cong Qian.

**Writing – original draft:** Han Wang.

**Writing – review & editing:** Han Wang, Haitao Hu, Jing Xu, Cong Qian.
